# Study on community structure of microbial consortium for the degradation of viscose fiber wastewater

**DOI:** 10.1186/s40643-017-0159-3

**Published:** 2017-07-10

**Authors:** Chao-Qun Ding, Kun-Rong Li, Yun-Xia Duan, Shi-Ru Jia, He-Xin Lv, He Bai, Cheng Zhong

**Affiliations:** 1 Key Laboratory of Industrial Fermentation Microbiology, (Ministry of Education), Tianjin University of Science & Technology, Tianjin, 300457 People’s Republic of China; 2 Tianjin Academy of Environmental Sciences, Tianjin, 300191 China; 3 CNOOC Ener Tech Beijing Research Institute of Engineering & Technology for Safety & Environmental Protection, Tianjin, 300457 China

**Keywords:** Viscose fiber wastewater, Bio-augmentation technology, Community structure, DGGE fingerprinting technology

## Abstract

**Background:**

Enrichment culture was applied to obtain microbial consortium from activated sludge samples collected from biodegradation system, a chemical fiber plant in Hebei Province, China. Bacterial composition and community dynamic variation were assessed employing denaturing gradient gel electrophoresis fingerprinting technology based on amplified 16S rRNA genes in the entire process of enrichment culture for viscose fiber wastewater.

**Results:**

Four bacteria named as VF01, VF02, VF03, and VF04 were isolated from the microbial consortium adopting the spray-plate method. The DNA bands of these four bacteria were corresponded to the predominant DNA bands in the electrophoresis pattern. VF01, VF02, VF03, and VF04 were phylogenetically closed to *Bacillus licheniformis*, *Bacillus subtilis*, *Paracoccus tibetensis*, and *Pseudomonas* sp. by sequence analysis, respectively. The degradation effects for COD_Cr_ of single isolated strain, mixed strains, and microbial consortium (VF) originally screened from viscose fiber wastewater were determined. The degradation ability was as follows: microbial consortium (VF) > mixed strains > single isolated strain. Microbial consortium (VF) showed the optimum degradation rate of COD_Cr_ of 87% on 14th day. Degradation of pollutants sped up by bio-augmentation of four strains. The molecular weight distribution of organic matter showed that viscose fiber wastewater contained a certain amount of large molecular organic matter, which could be decomposed into smaller molecular substances by microbial consortium (VF).

**Conclusions:**

The microbial consortium (VF) obtained from enrichment culture exhibited great potential for COD_Cr_ degradation. The screened strains had bio-augmentation functions and the addition of a mixture of four bacteria could speed up the degradation rate of pollutants.

## Background

Viscose fiber exhibits good physical, mechanical, and servicing qualities, and its status is next to polyester in chemical fiber industry (Lin 2000). Nowadays, a huge volume of wastewater has been produced in chemical industry which is responsible for serious environmental problems. Viscose fiber wastewater contains acid and alkaline wastewater. Acid wastewater is characterized by foul-smelling, high zinc ion concentration, high temperature, high salinity, sulfide, complex composition, and toxic substances. Alkaline wastewater is characterized by caustic soda and cellulose with low polymerization degree (Liang et al. 2009; Wang et al. 2013). The contaminants, as cellulose and lignin, in the wastewater are hard to be biodegraded and cause serious environment pollution, therefore it is very difficult to effectively deal with industrial wastewater (Kang and Yi 2005). These are the main reasons why the viscose fiber production has been kept stagnated over the past 20 years. Thus, it is of great social and environmental benefits to develop wastewater treatment technology.

The mixed wastewater of viscose fiber is mainly used in the industrial degradation. A large number of cellulose precipitate was formed after acid and alkaline wastewater were mixed (pH = 2–3) in the industrial degradation. At the same time, sulfide is turned into hydrogen sulfide and carbon disulfide in recycling (Liang et al. 2009). Zinc ions are turned into zinc hydroxide precipitation when the pH value was 8–9. Several physical and chemical methods have been used to treat wastewater and improve the degradation efficiency of COD_Cr_ (Hamaguchi et al. 2013; Kang et al. 2012). However, the cost is too high and the equipment covers a large area. In contrast, biological processes are the most environmental friendly and economical (Miao et al. 2009). However, no microorganism has been reported that can directly degrade viscose fiber wastewater, due to the complex composition of viscose fiber wastewater, most of which are cellulose, lignin, organic pollutants, and other large molecular substances (Liang et al. 2009; Vikman et al. 2002). It has been observed that few microorganisms can degrade macromolecular substance under high salinity condition (Kanaly and Harayama 2000).

Previous studies indicated that small molecular substances could be degraded by individual strain or single species (Kanaly and Harayama 2000; Head and Oleszkiewicz 2004). In contrast, large molecular substances should be degraded by diverse species of microorganisms, because the degradation could not be completed by individual type and the cooperation of diverse species is necessary (Kanaly et al. 2000). To study the degradation of viscose fiber wastewater by microorganisms, organic matter is converted through a range of metabolic reactions by assembling of microorganisms in wastewater environment.

It is possible to describe bacterial diversity in real environment by combining molecular biology technology based on 16S rRNA gene analysis and culture-dependent approaches as there have been a number of advances in microbial ecology. PCR–DGGE is a useful and powerful tool for describing the structure changes of microbial community in complex wastewater environments and enrichment cultures. PCR–DGGE has been widely used to identify the bacterial composition of different ecological niches, as these approaches are able to detect microorganisms which are not detected by culture-based methods (Muyzer and Smalla 1998; Brito et al. 2006). Molecular biology technology has contributed to further conduct the studies involving microbial composition, phylogeny, and metabolic mechanism during the whole wastewater treatment process (Al-Thukair et al. 2007; Widada et al. 2002).

The purpose of this paper was to (1) apply the viscose fiber wastewater enrichment strategy to establish viscose fiber wastewater degrading communities to enhance biodegradation of viscose fiber wastewater; (2) analyze the structure changes of bacterial community in viscose fiber wastewater by PCR–DGGE; (3) detect the biodegradability of bacterial consortium gathered from viscose fiber wastewater.

## Results and discussion

### DGGE analysis of community structure

**Fig. 1:**
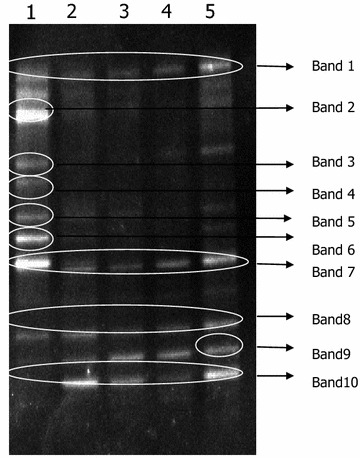
DGGE analysis of microbial community structure change under the selective pressure of viscose fiber wastewater (*Lanes 1* 1st day of subculture; *2* 10th day of subculture; *3* 20th day of subculture; *4* 30th day of subculture; *5* 40th day of subculture)

Ten bands were observed during enrichment process, indicating that the dynamic variation of bacterial community was complex. Figure 1 shows that the intensity of bands 2 and 6 were relatively high in the first lane (the first enrichment), while bands 2 and 6 were not found in the same position of other lanes. These results indicated that these two strains showed low degradation activities to contaminants in viscose fiber wastewater and were unaccommodated to high pressure. While the other strains showed high degradation activities to contaminants in viscose fiber wastewater and were accommodated to high pressure. During enrichment culture, bands 1 and 10 gradually turned bright, demonstrating that these two strains were the predominant microorganisms and showed strong abilities in degradation. Bands 3, 4, and 7 gradually turned dark and band 4 even could not be found in the later enrichment culture, demonstrating that the number of population tended to reduce during enrichment culture; however, some strains were still predominant for degradation. Bands 8 and 9 remained to be bright in all lanes during enrichment culture, suggesting that these strains might utilize organic matter in wastewater as C/N source.

### DGGE analysis of mixed culture

**Fig. 2:**
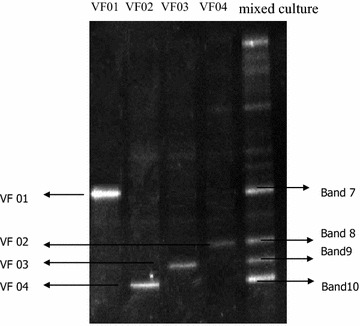
Analysis using DGGE and comparison of V3 fragments derived from enrichment culture and bacterial isolates (*lane VF01*–*VF04* isolates from the activated sludge; *Lane mixed culture* mixed culture at 14th day of subculture)

Four strains, VF01, VF02, VF03, and VF04, were isolated from the 5th enrichment culture. Bands in VF01, VF02, VF03, and VF04 were well correlated with the corresponding bands of the mixed culture in the PCR–DGGE (Fig. 2). In theory, DNA fragments corresponding to bands on the same horizontal line have identical sequence, the electrophoretic mobility of band 7 was identical with strain VF01; band 8 with strain VF02; band 9 with strain VF03; band 10 with strain VF04. These four bands existed in the whole enrichment cultures, confirming that these four isolate strains exhibited advantage in removing contaminants from wastewater compared with other isolated strains.

### Phylogenetic analysis

Bands 1, 2, 7, VF 01, VF 02, VF 03, and VF 04 were excised from gels following by identification of strains and analysis of phylogeny based on the 16S rRNA sequence (Figs. 1, 2). Bands 1, 2, and 7 were identified to be *Pseudomonas* sp. (191 bp, 99% identical), *Pseudomonas* sp. (201 bp, 99% identical), and *Bacillus* sp. (203 bp, 100% identical), respectively. Bands VF 01, VF 02, VF 03, and VF 04 were identified to be *Bacillus licheniformis* (1451 bp, 100% identical), *Bacillus subtilis* (1437 bp, 100% identical), *Paracoccus tibetensis* (1378 bp, 98% identical), and *Pseudomonas* sp. (1459 bp, 100% identical), respectively.

**Fig. 3:**
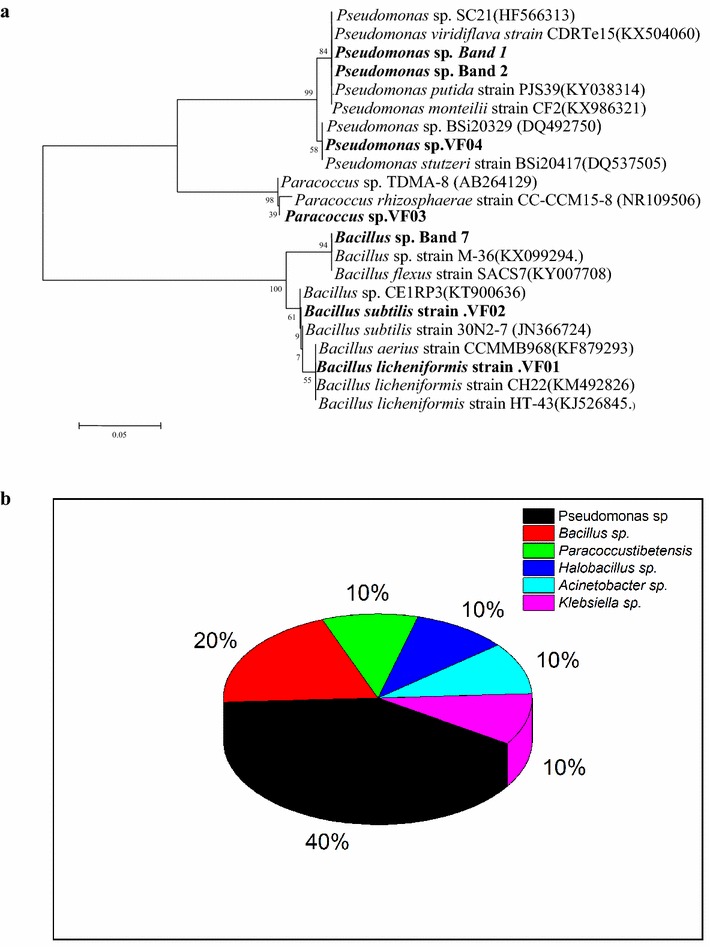
**a** Phylogenetic tree with cut bands: Band 1, Band 2, Band 7, Band 10; isolated strains: VF01, VF02, VF03, VF04; and their closest relatives derived from GenBank data based on 16S rRNA gene analysis. **b** The percentage of “community” in this kind of wastewater

Phylogenetic tree for these strains was developed (Fig. 3). VF01 and VF02 belonged to *Bacillus* sp., which was the common microorganism used for biodegradation and purification of industrial wastewater (e.g., *Bacillus pumilus*). *Bacillus* sp. had apparent advantage in degrading organic pollutants, such as organic phosphorus pesticides, petroleum pollutants, polycyclic aromatic hydrocarbons, phenol, and nitrobenzene (Devaraja et al. 2002; Oliveira et al. 2009; Gopinath et al. 2009; Singh et al. 2008; Wen et al. 2011). *Bacillus* sp. are widely distributed in the natural environment, some of which can grow well under high acid, high alkali, high temperature, and cold condition, making it possible to decompose complex polysaccharide, protein, and soluble organics.

VF03 was 98% identical with *P. tibetensis* sp., which is a kind of denitrification bacteria and can be separated from the wastewater plant. *P. tibetensis* sp. is mainly used in sewage treatment, such as landscape water treatment, urban river governance, especially aquaculture wastewater treatment.

VF04 was 99% identical with the *Pseudomonas* sp., which is widely distributed in the nature and has the ability to metabolize a series of compounds, such as polycyclic aromatic hydrocarbons, toluene, cyanide, carbazole, simple aromatic compounds, organic solvent in organic compounds, and chlorinated hydrocarbons (Reineke 1998).

### Degradation rate of COD_Cr_

**Fig. 4:**
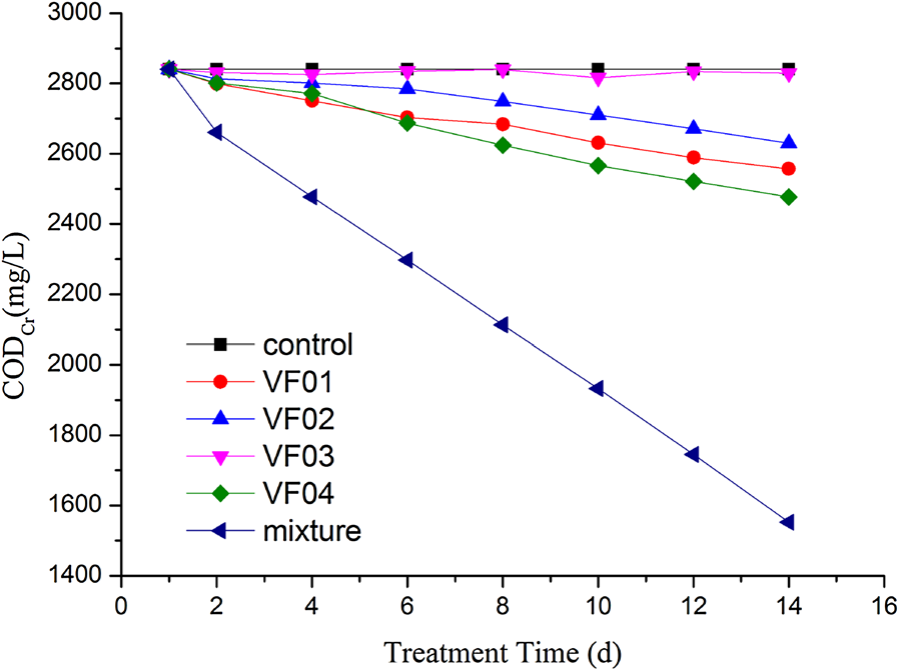
Biodegradation of COD_Cr_ by individual isolates, mixture of the four isolates, and the consortium VF

The degradation rate of COD_Cr_ of individual strains, mixed strains, and the enriched consortium (VF) were measured to better understand the metabolic capabilities of consortium. As shown in Fig. 4, after incubated for 14 days, strain VF01 could degrade 10% of COD_Cr_; strain VF02 could degrade 7.5% of COD_Cr_; and strain VF04 could degrade 14.8% of COD_Cr_; however, strain VF03 could not degrade COD_Cr_ in wastewater. The degradation effect of single strain was the worst, which ranged from 0.5 to 14.8%, as the viscose fiber wastewater contained complex macromolecular organic matter, such as cellulose, hemicellulose, and lignin. The degradation process involves in complex biochemical metabolic reaction and requires the synergistic action of different types of microorganisms. *Bacillus* sp. and *Pseudomonas* sp. could produce degradation enzymes of lignin and cellulose, making it possible to decompose cellulose, hemicellulose, and lignin in viscose fiber wastewater (Hernández et al. 2001; Tuomela et al. 2000; Kumar et al. 2001). However, the decomposition rate is very slow because the degradation enzymes are intracellular enzyme (Schwarz 2001). *P. tibetensis* sp. is a kind of denitrification bacteria, which cannot produce degradation enzymes but was able to convert nitrate to nitrogen and provided the nitrogen source for the growth of other organisms.

*Bacillus* sp. can cooperate with *Pseudomonas* sp. to degrade the large molecules substances in viscose fiber wastewater, such as cellulose and lignin, etc. (Tuomela et al. 2000; Kumar et al. 2001). The mechanism of synergistic degradation has been clear now (Wilson 2004). The interaction of fungi, bacteria, and the microbial community contributed to the complete degradation of large molecules (Hernández et al. 2001), but the degradation of single *Bacillus* sp. or single *Pseudomonas* sp. on cellulose, lignin, and other large molecules was limited.

**Fig. 5:**
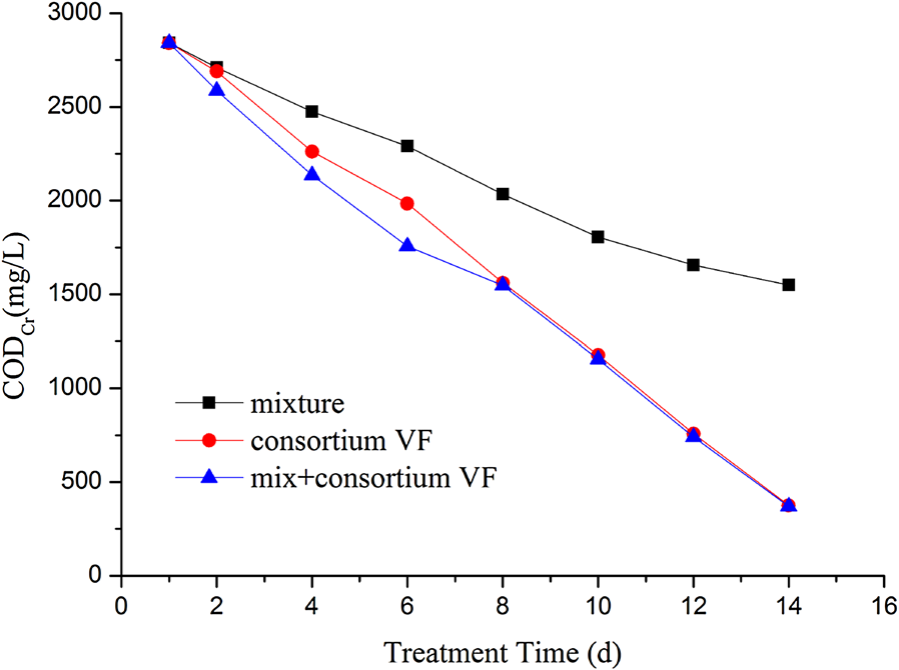
Biodegradation of COD_Cr_ by mixture of the four isolates, the consortium VF, and mixture + the consortium VF

The degradation rate of COD_Cr_ of the mixed strains, the enriched consortium (VF), and the mixture with consortium (VF) were measured to better understand the metabolic capability of the mixed strains. The degradation effect of COD_Cr_ depicted in Fig. 5 was as follows: consortium (VF) > the mixture of four isolates > individual isolates. This is due to the number and types of microorganisms in the consortium (VF) which are more than that of mixed and individual strains. The small molecular substances can be metabolized by individual strains, while the biodegradation of macromolecular substances requires the combined efforts of different strains, especially cellulose and lignin.

After operation for 14 days, the mixed strains degraded 45.4% of COD_Cr_, which was far below than 87% removal efficiency of consortium (VF), due to that the number and types of microorganisms in the consortium (VF) are more than that of mixture. In contrast, the degradation rate of the mixture and consortium (VF) is faster than that of consortium (VF) from the 1st to 8th day. After 8th day, the degradation rate of the mixture and the consortium (VF) was same with that of consortium (VF), indicating that partial large molecules could be degraded into small molecules by the mixture, such as cellulose and lignin, etc. The addition of the mixture in the consortium (VF) can improve the degradation effect on pollutants. *Bacillus* sp. and *Pseudomonas* sp. had bio-augmentation function and could enhance the degradation effect of viscose wastewater.

### Dissolved organic matters (DOM) size distribution in viscose fiber wastewater

**Fig. 6:**
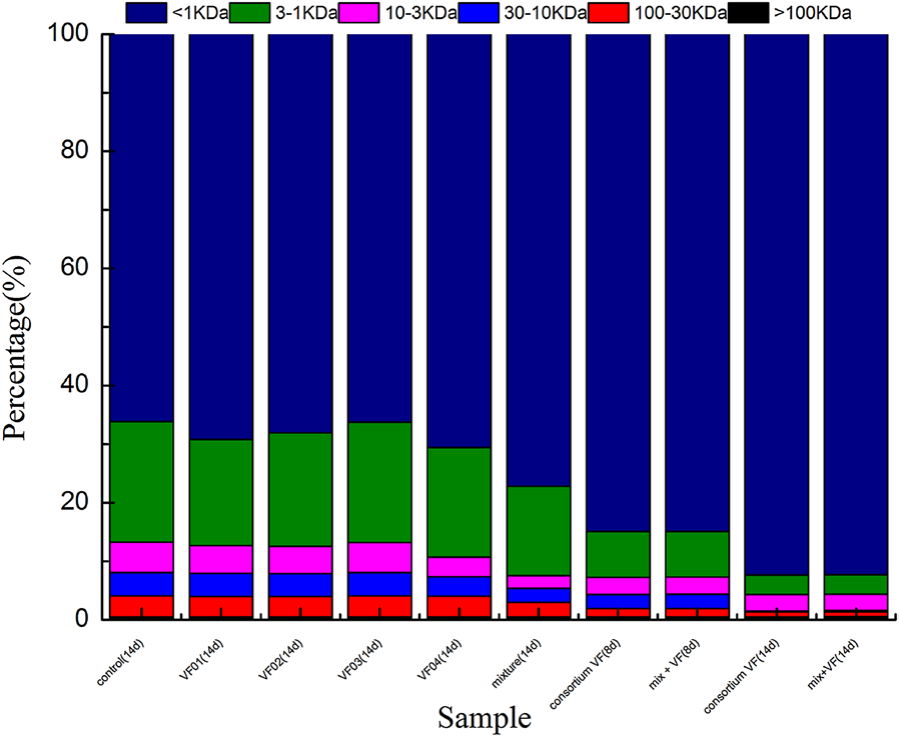
The molecular weight distribution and proportion of DOM in sewage biodegraded by different combinations of microorganisms for different days [*mix* + *VF* mixture with the consortium (VF)]

The molecular weight distribution of organic matter during the degradation process was measured to further analyze the bio-augmentation effect of four strains. As shown in Fig. 6, the molecular weight distribution in 10 samples was asymmetric and DOM was mainly composed of small molecules, e.g., <1 kDa. In control sample, the percentage of DOM (<1 kDa) reached 66.1% and the percentage of DOM (10-3 kDa) reached 20.6%. For large molecular organic matter, the percentage of DOM (>100 kDa, 100-30 kDa, and 30-10 kDa) reached 3.6, 4, and 5.2%, respectively. Viscose fiber wastewater also contained a lot of insoluble large molecular organics, thus leading to the difficulty of further degradation. The molecular weight distribution of VF01, VF02, VF03, and VF04 was similar to that of the control group. This result was consistent with the degradation effect of COD_Cr_ (Fig. 4).

Microbial degradation rate sped up by bio-augmentation of four strains by comparing the distribution of molecular weight. The organic compounds in wastewater were accelerated into small molecules by bio-augmentation of four strains. The proportion of the large molecular organic matter in wastewater became less and less, e.g., the percentage of 100-30 kDa changed from 3.6 to 0.85% and the percentage of 30-10 kDa changed from 4 to 0.25%. However, the fraction of small molecule organic matter became more and more, e.g., the percentage of <1 kDa changed from 66.1 to 92.27%. The molecular weight distribution of DOM in sewage not only reflected the characteristics of organic matter but also closely related to the degradation efficiency for wastewater (Chang et al. 2000).

Bio-augmentation utilizes microorganisms as bio-degraders to purify sewage in nature and bioreactors to remove contaminants (Chong et al. 1997; El Fantroussi and Agathos 2005; Head and Oleszkiewicz 2004; Reberto et al. 2003). Bio-augmentation is efficient in degrading target pollutants and removing the refractory organics involved in wastewater by inoculating strains. Previous studies indicated that bio-augmentation was feasible for the treatment of waste streams produced from pharmaceutical factories, coke plants, pulp mills, dye, and other industries (Saravanane et al. 2001; Park et al. 2008; Wang et al. 2002; Yu and Mohn 2001; Chen et al. 2006).

## Conclusions

The microbial consortium for wastewater degradation was enriched from samples of viscose fiber wastewater collected from a chemical fiber plant in Hebei, China. The method of PCR–DGGE was employed to investigate the changes in microbial community structure and helped us to determine the predominant species for COD_Cr_ biodegradation throughout the enrichment process. Consortium (VF) exhibited great potential in COD_Cr_ removal and the degradation rate of COD_Cr_ reached 87% until 14th day, indicating that enrichment culture as an isolation method was both feasible and effective. Four strains had biological reinforcement functions and the addition of a mixture of four bacteria could speed up the degradation rate of pollutants and the application of these species to bio-augmentation fields would assist in observing their catabolic behavior in highly polluted environments.

## Methods

### Media

Enrichment and cultivation were carried out in modified LB medium (Tryptone 5 g/L, yeast extract 1 g/L, NaCl 3 g/L, agar 1.5 g/L, pH 7.2–7.4).

### Sampling, enrichment, and isolation of the viscose fiber wastewater degraders

The mixed wastewater of viscose fiber samples were collected from a chemical fiber wastewater plant in Hebei, China. Activated sludge samples were collected from biochemical treatment system. The samples were stored at 4 °C until use. The characteristics of wastewater were listed in Table 1.

**Table 1:** The characteristics of viscose wastewater sample

Parameters	Value
COD_Cr_	2800–3000
BOD_5_	860–920
SS	1000–1200
TN	150–200
TP	75–85
TDS	24–30
pH	6–8
Zinc ion	300–500

The units for the parameters except for pH and salt are in mg/L, TDS is g/L

*ND* no detectable, *COD*_*Cr*_ chemical oxygen demand, *BOD*_*5*_ biochemical oxygen demand after 5 day, *SS* suspended solids, *TN* total nitrogen, *TP* total phosphorus, *TDS* salt

Activated sludge samples (2.0 g) were cultured in 250 mL Tri-flask with 100 mL LB media at 30 °C and 150 rpm for 2 days. Hereafter, this culture of 10 mL was shifted to another flask of 250 mL. The amount of viscose fiber wastewater was gradually increased until all wastewater was used as a microbial growth substrate at 10 days. The acclimation time for enrichment culture lasted for 40 days.

Bacteria were screened from the consortium by tenfold series dilution method. Morphologically distinct individual colonies were further purified on LB agar plates.

### DNA samples preparation

DNA was respectively extracted from the isolated strains and the enrichment cultures, i.e., the first day of enrichment culture sample, the 10th day of enrichment culture sample, the 20th day of enrichment culture sample, the 30th day of enrichment culture sample, the 40th day of enrichment culture sample following the manufacturer’s protocol (Qiagen, Valencia, CA, USA). The integrity of DNA was detected by agarose gel electrophoresis.

### Amplification of DNA and DGGE analysis

The variable region V3 of the 16S rRNA gene was amplified employing primers 341F (5′-CCT ACG GGA GGC AGC AG-3′) with a 40-bp GC clamp at 5′ end (CGC CCG CGC GCG CGG CGG GCG GGG CGG GGG CAC GGG GGG) and 518R (5′-ATT ACC GCG GCT GCT GG-3′) (Watanabe et al. 2001).

Cycling conditions for 16S rRNA gene amplification were as follows: initial denaturation at 94 °C for 5 min, 30 cycles each involved denaturation at 94 °C for 1 min; anneal at 55 °C for 1 min, extended at 72 °C for 1 min; and another extension at 72 °C for 10 min. 5 μL of PCR products was analyzed by electrophoresis on 2% agarose gel in 1× TAE buffer (20 mM Tris–HCl, 10 mM glacial acetic acid, 0.5 mM EDTA, pH 8.0) at 120 V for 30 min.

Samples of PCR products were loaded onto 8% (w/v) polyacrylamide gel within 30–60% denaturing gradient (100% denaturant consisted of 7 M urea and 40% deionized formamide). The electrophoresis was run in 1× TAE buffer for 10 h, (100 V, 60 °C) in Universal Mutation Detection System apparatus (Bio-Rad, Dcode, USA). Finally, the gels were stained with ethidium bromide solution of 0.1% (v/v) (1× TAE) for 15 min, rinsed in distilled water for 20 min, and then were scanned in a Gel Doc XR documentation system (Bio-Rad).

### Identification of strains and analysis of phylogeny

Pieces of DGGE bands were excised from polyacrylamide gel with sharp blade and then were, respectively, transferred to a new 2.0 mL Eppendorf tubes with 50 μL of sterilized ultra-pure water and incubated at 4 °C for a night to accelerate the diffusion of DNA out of the gel bands. The centrifugation was conducted at 10,000*g* for 6 min to obtain supernatant from above DNA solution. 1 μL supernatant was used as amplified template with a set of primer 341F and 518R with no GC clamp. Amplified products were further analyzed by the technique of denaturing gradient gel electrophoresis to confirm that they were successfully isolated.

Bacterial 16S rRNA genes were amplified employing primers 27F (5′V-AGA GTT TGA TCC TGGCTC AG-3′) and 1492R (5′-GGC TACCTT GTT ACG ACT T-3′) (Ikenaga et al. 2002). The amplification procedure was as follows: preliminary at 94 °C for 4 min; 30 cycles of 94 °C for 1 min, 55 °C for 1 min, 72 °C for 2 min, and a final extension at 72 °C for 10 min.

The amplified products were purified from agarose gel and bound with a pMD18-T vector and then transformed into *Escherichia coli* DH5 and the recombinant clones with a 1.5 kb insert were sent to be sequenced by sequencing company. All generated sequences were compared with nucleotide sequences of known sequences listed in GenBank databases (http://www.ncbi.nlm.nih.gov/) using BLAST (Basic Local Alignment Search Tool).The phylogenetic tree was constructed according to maximum-likelihood analysis implemented in MEGA6 software.

### Biodegradation of contaminants

Enrichment culture processes were monitored periodically to isolate the strains capable of removing COD_Cr_ in viscose fiber wastewater. Isolated strains presenting clear colonial morphology on LB agar culture medium were further purified and incubated in LB culture media of 200 mL at 30 °C. The cells were harvested by centrifugation at 12,000 rpm for 10 min when the optical density (600 nm) reached at 0.6, pellets were washed with sterile 0.85% NaCl (w/v) twice and then inoculated into conical flasks containing 300 mL of viscose fiber wastewater and placing on a rotary shaker (180 rpm) at 30 °C. The mixture of isolates, the consortium labeled VF, and the mixture with the consortium were added into viscose fiber wastewater under the same conditions. The culture flask containing 300 mL of wastewater without bacterial consortium (VF) was as control experiment. Flasks of the isolates, mixture, the consortium, and the control were taken out from the rotary shaker on the 0th, 2nd, 4th, 6th, 8th, 10th, 12th, and 14th days. The analysis of COD_Cr_ content in each sample was carried out with Microwave digestion COD analyzer (DRB-200).

The degradation rate of COD_Cr_ was calculated using the formula as follows:

$${\text{The degradation rate }} = \left( {1 - C_{\text{e}} /C_{\text{i}} } \right) * 100,$$

where *C*_i_ and *C*_e_ are raw water and the sample concentrations in mg L^−1^.

### The isolation of dissolved organic matters (DOM)

The relative molecular weight was determined by ultrafiltration membrane method to reflect the changes of dissolved organic matter (DOM) during the biodegradation process. Particulate matter in samples was firstly got rid of using 0.45 μm filtering membrane, pretreated samples were added into a continuous flow system consisting of UF following the method of Zhao et al. (2006). And the molecular weight range for each fraction is listed in Table 2.

**Table 2:** The corresponding molecular weight cut-off range of each fraction

Fraction	Molecular weight range
UF YM-100	Fraction 100,000–500,000
UF YM-30	Fraction 30,000–100,000
UF YM-10	Fraction 10,000–30,000
UF YM-3	Fraction 3000–10,000
UF YM-1	Fraction 1000–3000

The quantity of dissolved organic carbon (DOC) corresponding to each size fraction of DOM after filtered with different pore-size UF membranes was calculated from DOC concentration and corresponding volume.

The ultrafilter is Amicon 8200 type ultrafiltration cup which was produced by the United States Millipore, having an effective volume of 180 mL, effective film area of 28.7 cm^2^, maximum withstand pressure of 0.53 MPa, built-in magnetic stirring Pressure-driven high-purity nitrogen (Qiao et al. 2007).

### Analytical methods

The pH value was measured by a pH meter (METTLER TOLEDO, FE20K). The content of BOD_5_ was measured by incubation method. The content of total nitrogen (TN) and total phosphorus (TP) were analyzed using alkaline potassium persulfate digestion-UV spectrophotometer and molybdenum blue spectrophotometric (Carranzo 2012). Dissolved organic carbon (DOC) in water samples were measured using UV-per-sulfate technique and infrared carbon dioxide analyzer (Phoenix 8000), and calibrated with potassium hydrogen phthalate.

## References

[CR1] Al-Thukair AA, Abed RM, Mohamed L (2007). Microbial community of cyanobacteria mats in the intertidal zone of oil-polluted coast of Saudi Arabia. Mar Pollut Bull.

[CR2] Brito EMS, Guyoneaud R, Goñi-Urriza M, Ranchou-Peyruse A, Verbaere A, Crapez MAC, Wasserman JCA, Duran R (2006). Characterization of hydrocarbonoclastic bacterial communities from mangrove sediments in Guanabara Bay, Brazil. Res Microbial.

[CR3] Carranzo IV (2012) APHA, AWWA, WEF.”Standard methods for examination of water and wastewater. Anales De Hidrología Médica 5(2)

[CR4] Chang CN, Chao A, Lee FS (2000). Influence of molecular weight distribution of organic substances on the removal efficiency of DBPS in a conventional water treatment plant. Water Sci Technol.

[CR5] Chen BY, Chen SY, Lin MY, Chang JS (2006). Exploring bioaugmentation strategies for azo-dye decolorization using a mixed consortium of *Pseudomonas luteola* and *Escherichia coli*. Process Biochem.

[CR6] Chong NM, Pai SL, Chen CH (1997). Bioaugmentation of an activated sludge receiving pH shock loadings. Bioresour Technol.

[CR7] de Oliveira PL, Duarte MC, Ponezi AN, Durrant LR (2009). Use of *Bacillus pumilus* CBMAI 0008 and *Paenibacillus* sp. CBMAI 868 for colour removal from paper mill effluent. Braz J Microbiol.

[CR8] Devaraja TN, Yusoff FM, Shariff M (2002). Changes in bacterial populations and shrimp production in ponds treated with commercial microbial products. Aquaculture.

[CR9] El Fantroussi S, Agathos SN (2005). Is bioaugmentation a feasible strategy for pollutant removal and site remediation?. Cur Opin Microbiol.

[CR10] Gopinath KP, Murugesan S, Abraham J, Muthukumar K (2009). *Bacillus* sp. mutant for improved biodegradation of Congo red: random mutagenesis approach. Bioresour Technol.

[CR11] Hamaguchi M, Kautto J, Vakkilainen E (2013). Effects of hemicellulose extraction on the kraft pulp mill operation and energy use: review and case study with lignin removal. Chem Eng Res Des.

[CR12] Head MA, Oleszkiewicz JA (2004). Bioaugmentation for nitrification at cold temperatures. Water Res.

[CR13] Hernández M, Hernández-Coronado MJ, Ball AS, Arias ME (2001). Degradation of alkali-lignin residues from solid-state fermentation of wheat straw by streptomycetes. Biodegradation.

[CR14] Ikenaga M, Muraoka Y, Toyota K, Kimura M (2002). Community structure of the microbiota associated with nodal roots of rice plants along with the growth stages: estimation by PCR-RFLP analysis. Biol Fertil Soils.

[CR15] Kanaly RA, Harayama S (2000). Biodegradation of high-molecular-weight polycyclic aromatic hydrocarbons by bacteria. J Bacteriol.

[CR16] Kanaly RA, Bartha R, Watanabe K, Harayama S (2000). Rapid mineralization of benzo[a]pyrene by a microbial consortium growing on diesel fuel. Appl Environ Microbiol.

[CR17] Kang S, Li X, Fan J, Chang J (2012). Solid fuel production by hydrothermal carbonization of black liquor. Bioresour Technol.

[CR18] Kumar L, Rathore V, Srivastava H (2001). 14C-[lignin]-lignocellulose biodegradation by bacteria isolated from polluted soil. Indian J Exp Biol.

[CR19] Liang T, Dong-Ping LU, Bang HU, Zhang WL, Jiang LL (2009). Project design of viscose fiber wastewater treatment plant. China water & wastewater.

[CR20] Lin JH (2000). A new process for treatment of viscose fiber wastewater. China water & wastewater.

[CR21] Miao LH, Li FR, Wen JL (2009). Biological treatment of high-pH and high-concentration black liquor of cotton pulp by an immediate aerobic-anaerobic-aerobic process. Water Sci Technol.

[CR22] Muyzer G, Smalla K (1998). Application of denaturing gradient gel electrophoresis (DGGE) and temperature gradient gel electrophoresis (TGGE) in microbial ecology. Antonie Van Leeuwenhoek.

[CR23] Park D, Lee DS, Kim YM, Park JM (2008). Bioaugmentation of cyanide-degrading microorganisms in a full-scale cokes wastewater treatment facility. Bioresour Technol.

[CR24] Qiao C, Wei Q, Wang D, Yang M, Wei Q (2007). Molecular weight distribution and removal characters of DOM in the typical source water in south of China. J Acta Sci Circum.

[CR25] Reberto L, Vazquez SC, Mac Cormack WP (2003). Effectiveness of the natural bacterial flora, biostimulation and bioaugmentation on the bioremediation of a hydrocarbon contaminated Antarctic soil. Int Biodeter Biodeg.

[CR26] Reineke W (1998). Development of hybrid strains for the mineralization of chloroaromatics by patchwork assembly. Annu Rev Microbiol.

[CR27] Saravanane R, Murthy DVS, Krishnaiah K (2001). Bioaugmentation and treatment of cephalexin drug-based pharmaceutical effluent in an upflow anaerobic fluidized bed system. Bioresour Technol.

[CR28] Schwarz W (2001). The cellulosome and cellulose degradation by anaerobic bacteria. Appl Microbiol Biotechnol.

[CR29] Singh S, Chandra R, Patel DK, Reddy MM, Rai V (2008). Investigation of the biotransformation of pentachlorophenol and pulp paper mill effluent decolorisation by the bacterial strains in a mixed culture. Bioresour Technol.

[CR30] Tuomela M, Vikman M, Hatakka A, Itävaara M (2000). Biodegradation of lignin in a compost environment: a review. Bioresour Technol.

[CR31] Vikman M, Karjomaa S, Kapanen A, Wallenius K, Itävaara M (2002). The influence of lignin content and temperature on the biodegradation of lignocellulose in composting conditions. Appl Microbiol Biotechnol.

[CR32] Wang JL, Xiang CQ, Li BW, Yi Q, Hegemann W (2002). Bioaugmentation as a tool to enhance the removal of refractory compound in coke plant wastewater. Process Biochem.

[CR33] Wang X, Liu J, Huai-Bo GE (2013). Engineering of alkali recovery in cotton pulp black liquor treatment. China water & wastewater.

[CR34] Watanabe K, Kodama Y, Harayama S (2001). Design and evaluation of PCR primers to amplify bacterial 16S ribosomal DNA fragments used for community fingerprinting. J Microbiol Methods.

[CR35] Wen Y, Zhao G, Zhou C, Cao A (2011). Research progress of microbial agents in ecological engineering. Acta Ecol Sin.

[CR36] Kang Q, Yi S (2005). Industrialization test of using acid-out+ CASS+ air-floatation way to treat plasm-scum wastewater. Tech Equip Environ Pollut Control.

[CR37] Widada J, Nojiri H, Omori T (2002). Recent developments in molecular techniques for identification and monitoring of xenobiotic-degrading bacteria and their catabolic genes in bioremediation. Appl Microbiol Biotechnol.

[CR38] Wilson DB (2004). Studies of *Thermobifida fusca* plant cell wall degrading enzymes. Chem Rec.

[CR39] Yu Z, Mohn W (2001). Bioaugmentation with resin-acid-degrading bacteria enhances resin acid removal in sequencing batch reactors treating pulp mill effluents. Water Res.

[CR40] Zhao ZY, Gua JD, Fan XJ (2006). Molecular size distribution of dissolved organic matter in water of the Pearl River and trihalomethane formation characteristics with chlorine and chlorine dioxide treatments. Hazard Mater.

